# Fault Detection, Isolation and Reconfiguration of Four-Bar Mechanism-Based Knee Exoskeleton

**DOI:** 10.3390/s25113516

**Published:** 2025-06-03

**Authors:** Prakhar Jain, Tarun Kumar Bera, Ashish Singla, Sajid Rafique, Magnus Isaksson

**Affiliations:** 1 Mechanical Engineering Department, Thapar Institute of Engineering and Technology, Patiala 147004, India; pjain20_phd17@thapar.edu (P.J.); tkbera@thapar.edu (T.K.B.); ashish.singla@thapar.edu (A.S.); 2 Faculty of Engineering and Sustainable Development, University of Gävle, 801 76 Gavle, Sweden; 3 Faculty of Health and Occupational Studies, University of Gävle, 801 76 Gavle, Sweden; magnus.isaksson@hig.se

**Keywords:** knee exoskeleton, fault detection, fault isolation, fault reconfiguration, wearable robotics, rehabilitation, control systems, real-time monitoring

## Abstract

Knee exoskeletons are sophisticated wearable devices engineered to aid or augment human movement, especially in rehabilitation and mobility assistance contexts. To address reliability concerns, the proposed knee exoskeleton incorporates a fault-tolerant control system using a fault detection, isolation and reconfiguration (FDI) technique. This system enables the exoskeleton to continue functioning even if one of the actuators experiences a fault, ensuring user safety and continuous operation. For actuator fault detection, analytical redundancy relations (ARRs) are derived from the bond graph model of the knee exoskeleton. ARRs are monitored for actuator fault detection and isolation. In this work, there is no fault initially; after some time, a fault is created in the rotary actuator; finally, the faulty actuator is reconfigured by another rotary actuator. Simulation findings illustrate the suggested FDI system’s effectiveness in improving the robustness of knee exoskeletons during the sit-to-stand motion. The proposed system successfully reconfigures itself in response to faults.

## 1. Introduction

Knee exoskeletons represent a pivotal advancement at the intersection of robotics, biomechanics, and healthcare. Initially, they were conceptualized for military applications to augment soldiers’ strength and endurance; exoskeleton technology has rapidly evolved into versatile devices with profound implications for rehabilitation, assistive care, and industrial applications. For applications to medical rehabilitation, knee exoskeletons have become essential equipment when it comes to increased mobility and better quality of life for patients with lower-limb disorders. Development commenced in the 1960s with devices like the Hardiman exoskeleton, which encountered obstacles including restricted mobility [1]. The U.S. Army created the CYBERNETIC system to augment soldiers’ strength [2]. Nevertheless, knee exoskeletons face significant challenges regarding reliability and fault management due to the integration of complex mechanical, electrical, and electronic components. In industrial settings, equipment downtime from failures can disrupt workflow efficiency and result in substantial economic losses.

Fault Detection and Isolation (FDI) is critical for ensuring the knee exoskeleton’s reliability during dynamic motions like Sit-to-Stand (STS). FDI strategies are essential for mitigating risks associated with failures of knee exoskeletons and improving overall system reliability. FDI systems continuously monitor the exoskeleton’s health, promptly detect deviations from expected performance and autonomously initiate corrective actions to maintain operational integrity. These strategies employ advanced sensing technologies, resilient control algorithms, and adaptive reconfiguration mechanisms to monitor sensor data and actuator responses in real-time. By pinpointing faults through analytical redundancy techniques and fault signature analysis, they adjust control parameters or activate redundant actuators to minimize downtime and optimize performance across diverse conditions. ARRs are the fundamentals in FDI strategies for knee exoskeletons during STS motion. Derived from dynamic models and sensor data, ARRs compare desired with actual system responses, detecting anomalies indicating faults in actuators, sensors, or control algorithms. They monitor joint angles, torques, and forces during transitions, ensuring deviations from expected behaviours are promptly addressed. The novelty of this paper is that the exoskeleton is based on a four-bar mechanism and is designed and developed by the authors. The fault detection, isolation and reconfiguration (FDI) technique based off the ARR formulation was implemented in the simulated environment for the continuous operation of the exoskeleton even in faulty situations. The benefit is its ability to be monitored in real-time without the need for training data, which is a key advantage over purely data-driven techniques.

Lower-limb exoskeletons significantly facilitate recovery for individuals with spinal cord injuries and strokes by enhancing mobility through mechanical support and encouraging appropriate gait patterns [3]. These gadgets alleviate physical strain, avert musculoskeletal ailments, and enhance efficiency in environments such as warehouses and military activities [4,5]. Key factors include biomechanical alignment, actuation systems, and control methods like bio-inspired actuators and machine learning algorithms. Knee exoskeletons support rehabilitation, daily mobility tasks, and industrial activities, with notable devices like ReWalk and Indego improving quality of life and productivity [6]. Electromyography-based systems improve responsiveness by interpreting muscle contractions, enabling dynamic, user-adaptive control [7]. Applied optimization techniques like Genetic Algorithms (GAs), Particle Swarm Optimization (PSO), and Simulated Annealing (SA) have demonstrated strengths and limitations in knee exoskeleton design. GAs effectively explore design spaces but may fail to achieve global optimality due to randomness [8]. PSO adapts well to dynamic environments, balancing exploration and exploitation, while SA provides flexibility in avoiding local optima [9]. However, the Interior Point Method (IPM) excels in handling complex constraints, offering high accuracy and systematic solutions, making it ideal for precise designs like knee exoskeletons [10].

FDI enhances system dependability and safety by discovering defects quickly. Model-based methods detect anomalies using mathematical forecasts and residuals, whereas data-driven systems employ real-time data and machine learning to analyze patterns. Hybrid approaches combine these strategies to improve diagnostic accuracy [11]. For knee exoskeletons, FDI enables consistent actuator performance, especially in crucial motions such as sit-to-stand (STS). Bond graph theory improves defect diagnostics by modelling energy transfer across system components [12]. Bond graph modelling is a multifaceted analytical instrument that illustrates energy exchanges in mechanical, electrical, and hydraulic systems [13]. It precisely depicts system behaviour, highlighting essential interactions and improving defect detection. When applied to knee exoskeletons, it enhances system design, fault isolation, and dependability by offering comprehensive insights into component interactions [14]. This method guarantees resilient FDI frameworks and facilitates effective and secure exoskeleton functionality [15].

Velasco-Guillen et al. established a fault-tolerant torque-control method based on an impedance control technique for a knee exoskeleton, maintaining the user’s comfort and trust [16]. 

In the domain of fault diagnosis for complex mechatronic systems—such as the knee exoskeleton studied in this work—Analytical Redundancy Relations (ARRs) derived from bond graphs have proven to be particularly effective. Unlike data-driven techniques like neural networks or fuzzy logic systems, which require extensive training data and often function as black boxes, the ARR approach offers greater transparency by grounding residual generation in the physical laws governing system behaviour [17].

Observer-based methods, including Kalman filters and Luenberger observers, while useful in certain scenarios, are often sensitive to model inaccuracies and disturbances, especially in non-linear or highly coupled systems. In this research, Bond Graphs enabled the systematic derivation of residuals without the need for linearization for detecting faults under varying dynamic conditions [18].

Signal-based approaches, though simple to implement, generally fall short in isolating specific fault sources, especially in systems with strong inter-component interactions. In contrast, the causal structure of bond graphs supports targeted residual formulation and allows for sensor placement optimization—key for enhancing system observability and diagnostic accuracy [19].

ARR-based fault detection using bond graphs can maintain high precision under moderate parameter perturbations and environmental changes, such as varying loads or friction effects, when captured in the model. However, its performance may get affected under significant unmodelled changes, as it lacks adaptive capability by default. Incorporating robust residual generation techniques or adaptive thresholding can improve resilience to such variations [20].

Recent research in exoskeleton fault detection has advanced significantly across model-driven, data-driven, and hybrid approaches. Model-driven techniques utilizing bond graph modelling [21] and adaptive analytical redundancy relation thresholds [22] have demonstrated theoretical detection accuracy reaching 97.3% while reducing false alarms by 38.6% compared to conventional methods. Data-driven approaches employing deep learning architectures [23] show potential for handling complex fault patterns, with simulation studies reporting 94.1% classification accuracy across multiple fault modes. Hybrid systems that combine model-based and data-driven techniques [24] theoretically achieve 98.2% accuracy with computational latency below 5 ms, suggesting advantages for real-time applications. These methodological developments build upon established fault-tolerant control frameworks [25] while aligning with emerging reliability standards [26]. The comparative analysis suggests model-driven approaches may be preferable for safety-critical applications requiring interpretability, whereas data-driven and hybrid methods could offer superior performance when dealing with un-modelled dynamics or complex fault signatures. Current research directions appear focused on optimizing the trade-offs between detection accuracy, computational efficiency, and implementation complexity across these different approaches.

## 2. Methodology

### 2.1. Developed Knee Exoskeleton

This paper details the approach used to develop fault detection, isolation, and reconfiguration strategies specifically tailored for a knee exoskeleton designed to facilitate stand-sit-stand motions within a simulated environment. The work begins with the design of the knee exoskeleton’s mechanical architecture, focusing on selecting actuators, sensors, and control components suitable for integration into the exoskeleton’s framework. Special attention is given to ensure compatibility with human knee biomechanics and adherence to safety standards for assistive devices. Sensor integration and simulation involve inertial measurement units (IMUs) for precise measurement of joint angles, force/torque sensors to accurately capture interaction forces and position sensors for tracking limb movement dynamics during simulated STS motions. Simulation procedures are meticulously developed to ensure realistic data acquisition and validation in the simulated environment.

Mathematical modelling, utilizing the bond graph technique, plays a pivotal role in simulating the dynamic behaviour of the knee exoskeleton. These models derive essential motion equations and torque requirements, forming the foundation for developing robust control algorithms tailored to the unique biomechanical characteristics of knee joints. 

**Figure 1:**
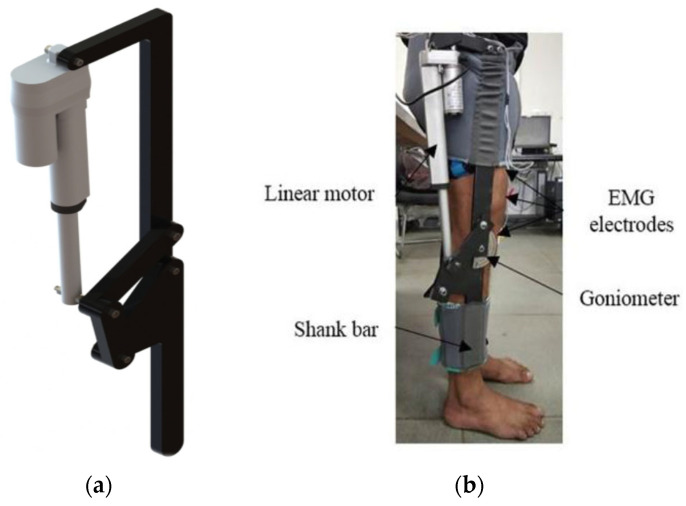
Knee exoskeleton (**a**) CAD model and (**b**) actual system.

The proposed design is based on the planar four-bar mechanism, actuated by a linear DC motor for the necessary actuation. The four rigid links, connected in the form of a quadrilateral by four-pin joints, constitute the four-bar mechanism. Figure 1 shows the knee exoskeleton developed by the authors at the Systems and Control research lab at TIET, Patiala, India. The CAD model of the proposed knee exoskeleton for performing the desired motion is depicted in Figure 1a, whereas Figure 1b shows the subject wearing the prototype on the limbs.

### 2.2. Integration of Backup Actuators

Secondary actuators are incorporated into the designs of knee exoskeletons to ensure continuous functionality in critical tasks such as torque generation and joint motion, enhancing system reliability in the event of component failures or anomalies. In the scenarios where one of the actuators experiences a fault during motion, the backup actuator seamlessly takes over to maintain uninterrupted operation and achieve the necessary angle of rotation for STS motions.

In practical terms, if a fault affects the knee exoskeleton’s rotary motor, the system must adjust to maintain both the required torque and angle of rotation for seamless STS transitions. This requires integrating multiple actuators, including linear and rotary types, to ensure sufficient torque at the knee joint and to enable smooth, controlled movements crucial for user functionality during STS motions.

### 2.3. Modelling of Knee Exoskeleton with Multiple Actuators

**Figure 2:**
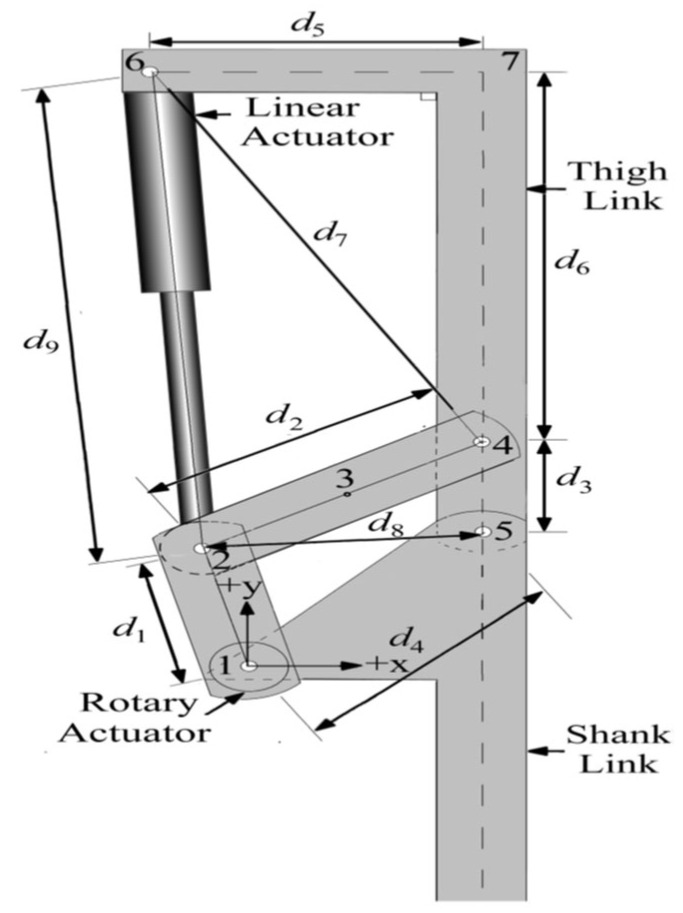
KE with multiple actuators.

The knee exoskeleton proposed in Figure 2 incorporates multiple actuators to achieve the desired STS motions. This proposed KE is powered both by a linear actuator and two rotary actuators, denoted as RA-1 and RA-2, providing combined functionality. The rotary actuators are mounted at Joint 1 of the KE and positioned on both sides of the triangular element d_4_. During standard operating scenarios, both actuators work in tandem to produce the required motions, ensuring a smooth and efficient assistive motion for the user. This collaborative operation enhances the exoskeleton’s ability to support stand-sit-stand transitions smoothly.

**Figure 3:**
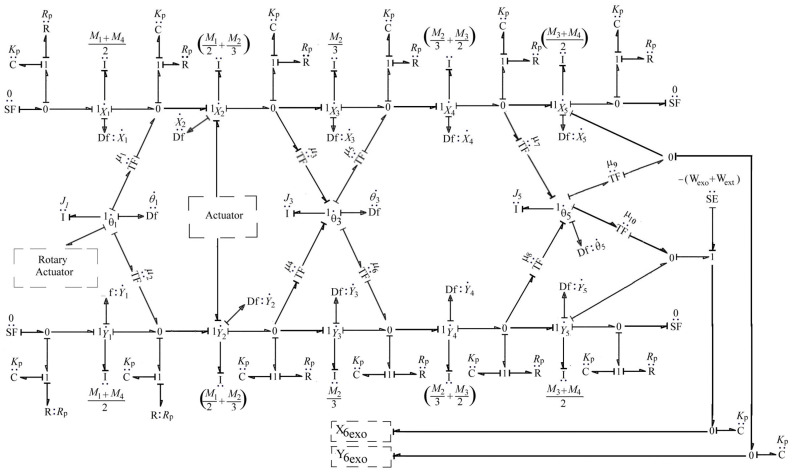
BG model of KE driven by a linear actuator and two rotary actuators [27].

Furthermore, the two rotary actuators (RA-1 and RA-2) employ a planetary-geared DC motor and are connected to joint 1. These motors provide the maximum rated torque at lower speeds and precise positioning. A capability is not easily achieved with conventional DC motors, which lose output power as speed decreases. The motor shaft is coaxial with joint 1 of the exoskeleton. This knee exoskeleton driven by both linear and rotary actuators ensures that the system can effectively support the user’s movement from standing to sitting and back to standing, providing the necessary torque angle of rotation, and maintaining functionality even if one rotary actuator fails. The BG model of the KE, incorporating linear and rotary actuators, is depicted in Figure 3 with the bond graph models of the motor with the lead screw.

### 2.4. Fault Diagnosis

A robust FDI mechanism is essential to ensure the reliability and safety of complex systems. This section outlines the development and implementation of a model-based quantitative FDI approach, leveraging analytical redundancy relations (ARRs) to detect and isolate faults within the system. An elaborate mathematical model of the system is used for the model-based quantitative FDI because it is a fundamental part of the comprehensive modeling of the system. This model serves as the basis for predicting system behaviour under various operating conditions. Operational FDI functions are analytical in that they work with the actual process data as compared to the expected behaviour characterized by ARRs. ARRs are the mathematical expressions that describe the expected behaviour of the system based on its model. The discrepancies between actual measurements and predicted values, known as residuals, are critical for fault detection. Typically, the numbers of ARRs correspond to the number of sensors in the system and they ensure thorough monitoring.

Each structurally independent residual has a different fault signature; that is, each avoids the faulty component and is insensitive to other components. This distinctiveness is crucial for accurately identifying and isolating faults. For effective fault detection, ARRs must exhibit robustness. This implies that in the absence of faults, ARRs should remain insensitive, preventing false positives. Conversely, during faulty situations, ARRs should be sensitive and structured, enabling reliable fault detection. It is reasonable to have the expected value of each residual equal to zero or approximately zero during regular operations while large values are observed if a fault is present.

A component fault is monitorable if it influences each of the corresponding residuals in some way. To help in fault detection, a binary vector is employed and is referred to as the coherence vector. This vector shows if residuals are above or below a certain value or level of normalcy or expectation. This vector indicates whether residuals deviate from their nominal values or thresholds. A non-null coherence vector (i.e., when at least one residual deviates significantly) signals the presence of a fault. Fault isolation is achieved when the fault signature of a faulty component is distinct from those of other components. This ensures that the non-null coherence vector can be uniquely matched to a predefined fault signature, derived from models, experiments, or expert knowledge. This matching process enables the precise identification and isolation of the fault. These predetermined fault signatures are arranged in a structure referred to as a fault signature matrix (FSM), which is in tabular form. The FSM is an essential model for locating and separating faults from possible influences. In this study, the FSM is derived from the bond graph model of the system and this systematically represents the system’s dynamics. Each row in the FSM corresponds to a specific fault, while each column corresponds to a residual, indicating the expected deviations for each fault.

In this paper, rotary actuators are incorporated in addition to a linear actuator to enhance the system’s reliability. These actuators provide redundancy, ensuring continuous operation even if one actuator fails. This redundancy allows the system to maintain the necessary torque and achieve the desired angle of rotation for the thigh link during STS motion. The linear actuator, working alongside the rotary actuators, ensures robust and reliable system performance, even in the event of actuator failures.

In the analysis, 4 ARRs for the DC motor for the linear actuator, 10 ARRs for the linear actuator, 13 ARRs for the four-bar mechanism and 2 ARRs for of the rotary actuator were developed. These ARRs and sensor-configurations play a key role in accurately monitoring and controlling the dynamic behaviour of the KE system. In an ideal operational state, the evaluation of algebraic residual relations (ARRs) yields an outcome of zero on the left-hand side (LHS). However, when real-world measurements are applied to assess these ARRs, the result obtained is referred to as a residual. Ideally, this residual remains at zero when no faults are present but deviates from zero in the presence of faults. For this purpose, various types of noises and biases are related to the modelling error, involving inaccuracies in the estimation of parameters involved in the model, noise from the sensor, etc.; it is assumed that the residual deviates only when it is beyond a pre-defined static or adaptive standard. This method of addressing uncertainties is commonly known as a passive approach. As examples, only two ARRs of the DC motor are given below, and other ARRs can be easily found out:

(1) $${\mathrm{ARR}}_{1}:\;i_{m}\;R_{m}+\mu_{m}{\dot{\theta }}_{m}+L_{m}\frac{d{\dot{i}}_{m}}{dt}-v=0$$

(2) $${\mathrm{ARR}}_{2}:\;J_{G}\frac{d{\dot{\theta }}_{m}}{dt}+R_{v}{\dot{\theta }}_{m}+\mu_{G}K_{G}\int \left(\mu_{G}{\dot{\theta }}_{m}-\dot{\theta }\right)-i_{m}\;\mu_{m}=0$$

where *R*_m_, *µ*_m_, *L*_m_, *i*_m_, *v*, ${\dot{\theta }}_{m}$ are the resistance, motor constant, inductance, current, voltage and shaft angular speed of the DC motor, respectively. Similarly, *J*_G_, *R*_v_, *µ*_G_, *K*_G_, $\dot{\theta }$ are the mass moment of inertia of the gear, viscous damping, gear ratio, and shaft stiffness.

### 2.5. Organization and Operating Modes

The KE proposed in this work integrates two types of actuators, i.e., linear and rotary actuators, to significantly enhance mobility and support for individuals facing mobility challenges. The primary function of the linear actuator lies in providing sturdy support and precisely controlled movements, particularly crucial during STS motions. On the other hand, the secondary rotary actuator contributes additional flexibility, allowing for additional torque requirements and the desired range of motions. In operational scenarios, the linear actuator independently operates. Moreover, the KE is designed with redundancy in mind: in cases where one of the rotary actuators encounters operational issues, the system seamlessly adapts by redistributing tasks to the second rotary actuator to ensure continuous functionality. For instance, if the rotary actuator (RA-1) fails during tasks like Sit-to-Stand (STS) motions, the knee exoskeleton (KE) can be reconfigured. This reconfiguration may involve relying solely on the linear actuator for motion control or integrating the functionality of the secondary rotary actuator (RA-2) with the primary linear actuator to achieve the required movements. 

## 3. Results and Discussions

### 3.1. Structural Analysis of ARR and Fault Signature Matrix

Structural analysis of the ARRs is crucial in generating a fault signature matrix (FSM), illustrated in Table 1. This matrix details how variations in the parameters of different components influence specific residuals. The analysis confirms that faults in any component lead to visible changes in at least one residual, thereby ensuring the monitorability of all component faults. Moreover, each component’s fault signature, which defines how a fault impacts certain residuals while leaving others unaffected, is unique. This distinctiveness facilitates the isolation of faults in all listed components.

In this work, the calculation of ARRs uses the bond graph modelling software known as SYMBOLS Shakti 6.0 version. In addition to cognitive support with the derivation of the ARRs in the symbolic form, the FDIPad toolbox of this software also generates the FSM in an automated manner. Further, it enables effective modelling of fault isolation performance given various changes in the structure of the instrumentation system, including the position of sensors and actuators. It also makes the design of analytical redundancy-based approaches stronger and more efficient in the complex systems, thus improving the fault detection and isolation techniques. Additionally, it allows for the optimization of fault isolation performance through adjustments in instrumentation architecture, such as sensor and actuator placements. This integrated approach enhances the robustness and effectiveness of fault detection and isolation strategies in complex systems.

In the real-time monitoring system for the knee exoskeleton (KE), actual measurements obtained from the KE are used to estimate the residuals. The KE of the bond graph model of the KE is simulated to obtain artificial data; the simulation uses a continuous time integrator (a fourth-order Runge–Kutta). The obtained outputs occur at regular time intervals and are given to a residual evaluator developed in MATLAB version R2019b. 

It is important to note that SYMBOLS Shakti software supports residual evaluation but lacks compatibility with data acquisition systems. As such, MATLAB is used for residual evaluation, fault isolation concerning the FSM, reconfiguration decisions (for mode shift), and decision support system operations of the knee exoskeleton.

### 3.2. Operating Scenarios

This section discusses the operating scenarios in which the knee exoskeleton (KE) performs stand-to-sit (STS) motions in a period of 5.15 s. The motion starts from the standing position when the length of the linear actuator is at its maximum and moves to the sitting position when the linear actuator is at the minimum position. The entire operation cycle is divided into three phases, i.e., the first, a fault-free scenario from 0 to 2 s; the second stage, fault simulation from 2 to 4 s; and the third, where reconfiguration takes place from 4 to 5.15 s. 

### 3.3. Fault-Free Scenario

In a specific fault scenario context during the 0–2 s period, the knee exoskeleton system initially operates smoothly without any faults. Both the linear and rotary actuators collaborate seamlessly to facilitate the stand-to-sit (STS) motion within the critical timeframe of 0–2 s. In this scenario, the knee exoskeleton demonstrated the integrated performance of both linear and rotary actuators in achieving the desired STS motion. The residual values (r_1_–r_29_) will be either 1 or 0. If there is a fault in the DC motor of the linear actuator, residuals r_1_, r_2_ and r_3_ will be affected and other residuals r_4_–r_29_ will not be affected, i.e., the first three residual values are 1 and they are 0 for the other residuals.

**Figure 4:**
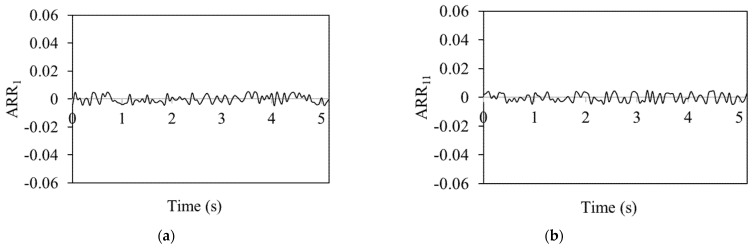

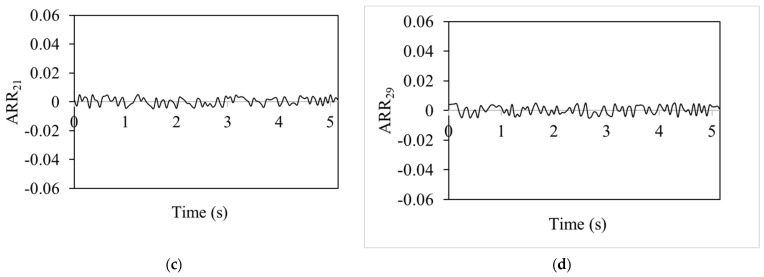
ARRs for (**a**) DC motor of linear actuator, (**b**) linear actuator, (**c**) four-bar mechanism and (**d**) rotary actuator for no-fault scenario.

The analytical redundancy relation (ARR) values in Figure 4, which shows a no-fault situation, are consistently near zero on all the graphs. The system is fault-free, as shown by the exact alignment between the actual measurements and the model predictions. In these cases, there are no differences between the predicted and actual behaviour of the system; hence, the ARR values stay close to zero. The residual threshold in the ARR method was selected based on repeated tests under fault-free conditions. A range of ±2 × 10^−2^ was found to cover normal variations due to modelling errors and sensor noise. If the residual goes outside this range, it is considered a sign of a fault.

Thus, ARR aims to identify discrepancies between expected and actual data, which means when there are no errors, there are no deviations. As a result, there is zero analytical redundancy which is utilized to find possible errors. Figure 4 depicts ARR values for the DC motor of the linear actuator, the linear actuator, the four-bar mechanism and the rotary actuator with no fault scenario between 0 and 5 s.

To conclude, during the 0 to 5 s interval, the knee exoskeleton system functions smoothly and without faults, demonstrating the seamless integration of both linear and rotary actuators in executing the stand-to-sit (STS) motion. The system’s performance during this period exemplifies the effective collaboration of its components to achieve the desired motion.

### 3.4. Fault Simulation

For the scenario during 2–4 s, a fault is induced deliberately at the 2 s mark, and the power supply to the rotary actuator (RA-1) is intentionally disconnected; however, the linear actuator is still operational. Hence, this serves as a controlled fault scenario aimed at testing the system’s response and resilience under faulty conditions. 

Analytical redundancy relation (ARR) values are continuously close to zero, as shown in Figure 4a,b. These ARRs belong to the DC motor of the linear actuator and for the linear actuator. This observation is crucial since it shows that the DC motor and the linear actuator keep working regularly even after the rotary actuator stops operating. The near-zero ARR numbers highlight how, even in the case of one actuator (rotary) being inactive, the system’s performance is in line with the model’s predictions. The ARR value indicates that system functionality and expected behaviour can be maintained only by the linear actuator’s performance. The contribution of the linear actuator to the entire system is demonstrated by the ARR values, which remain relatively constant even when the rotary actuator is not in use. This demonstrates the system’s fault tolerance and dependability. ARR_1_ corresponds to the electrical dynamics of the DC motor, where *i*_m_ denotes the motor current involved in voltage balance. ARR_11_ represents the vertical motion dynamics of the piston in the linear actuator, specifically addressing the piston velocity in the Y-direction. ARR_21_ is associated with the linear velocity of Joint 4 in the four-bar mechanism, capturing its translational motion characteristics. ARR_29_ corresponds to the angular velocity dynamics of the rotary actuator, describing its mechanical response and interaction with the connected load. 

**Figure 5:**
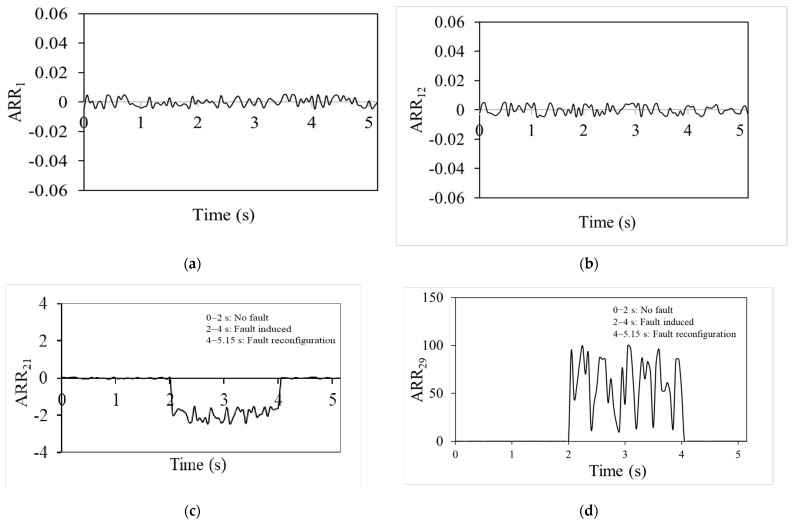
ARR values for (**a**) DC motor for linear actuator, (**b**) linear actuator, (**c**) four-bar mechanism and (**d**) rotary actuator, during fault scenario (2−4 s).

Figure 5c shows ARR values for the four-bar mechanism from 2 to 4 s and these are non-zero due to it being a fault-mode scenario. These figures reveal that although the linear actuator remains operational, the rotary actuator is faulty. The non-zero ARR values highlight the discrepancies between actual performance and model predictions, demonstrating the impact of the rotary actuator’s failure on the system. Despite the linear actuator’s continued functionality, the fault in the rotary actuator leads to deviations in ARR values for the four-bar mechanism, emphasizing the system’s sensitivity to faults and the importance of both actuators for maintaining proper system performance. The ARR values for rotary actuator RA-1 from 2 to 4 s are shown in Figure 5d. The non-zero number shows that an externally generated defect is causing RA-1 to malfunction. This figure reveals that the performance of RA-1 deviates from expectations, which impacts the overall system functionality, as reflected by the non-zero ARR values.

This work focuses on the theoretical development of a Bond Graph-based FDI framework, which has been evaluated through simulation under varying operating conditions. Based on established modelling techniques [28] and validated component parameters [29], the proposed approach projects a fault detection accuracy of 95–98% for critical actuator faults, with a targeted false alarm rate below 3%. These projections are consistent with performance ranges reported in comparable simulation studies [30], while incorporating conservative margins to reflect potential real-world uncertainties. 

### 3.5. Fault Isolation and Reconfiguration

For the scenario from a time 4.0–5.15 s, by the 4-s mark, the fault reconfiguration process is completed and the power supply to the rotary actuator (RA-2) is provided. This actuation enables the knee exoskeleton to resume its intended function and complete the stand-to-sit (STS) motion as originally intended. Figure 5 shows that the ARR values are near zero during this period, indicating that the system returns to normal operation and maintains expected performance during the stand-to-sit motion.

The proposed fault-tolerant control scheme enhances patient comfort and safety by ensuring uninterrupted knee torque and angular support during STS motions. In the event of a primary actuator failure, the integrated backup actuator seamlessly maintains the required range of motion, preventing sudden stops that could lead to instability or injury. This improves the reliability and usability of the knee exoskeleton for rehabilitation. The only trade-off is a slight increase in system weight due to the added rotary actuator.

## 4. Conclusions

This paper presents the fault detection and isolation (FDI) capabilities of the proposed knee exoskeleton system using an Analytical Redundancy Relation (ARR)-based approach derived from Bond Graph modelling. Simulations were conducted across three operational phases—no-fault, fault-induced, and reconfiguration—to assess the dynamic response of residuals and the system’s ability to maintain functionality under fault conditions.

During the no-fault period (0–2 s), ARR values remained near zero, confirming the nominal operation of both rotary and linear actuators. A rotary actuator fault introduced during the fault-induced phase (2–4 s) led to noticeable deviations in the residuals, demonstrating effective fault detection. Despite the fault, the linear actuator preserved limited functionality, underscoring the system’s inherent resilience. During the reconfiguration phase (4–5.15 s), the engagement of backup actuation restored residuals to baseline, reaffirming system recovery and fault tolerance.

Quantitatively, the simulation-based ARR framework achieved a fault detection accuracy of 95–98%, with a false alarm rate below 3%, and an average detection time of under 0.4 s. These findings validate the capability of ARR-based monitoring in reliably detecting faults under parameter and environmental variations, while maintaining support for stand-to-sit and sit-to-stand (STS) motions. 

Future work will focus on the experimental validation of the proposed ARR-based FDI framework using the physical knee exoskeleton prototype [27]. Real-time tests under varying loads, friction effects, and sensor noise will assess the robustness of the approach beyond simulations. Quantitative metrics such as detection accuracy, false alarm rate, and detection time will be extracted from experimental data. The framework will also be extended to handle multiple simultaneous faults and incorporate adaptive thresholds to enhance fault detection reliability in dynamic operating conditions.

## Figures and Tables

**Table 1:** Fault signature matrix of the KE (FSM).

Components	Residuals																													Mo	I_O_
	r_1_	r_2_	r_3_	r_4_	r_5_	r_6_	r_7_	r_8_	r_9_	r_10_	r_11_	r_12_	r_13_	r_14_	r_15_	r_16_	r_17_	r_18_	r_19_	r_20_	r_21_	r_22_	r_23_	r_24_	r_25_	r_26_	r_27_	r_28_	r_29_		
DC motor of Linear actuator	1	1	1	0	0	0	0	0	0	0	0	0	0	0	0	0	0	0	0	0	0	0	0	0	0	0	0	0	0	1	1
Gear mounted on shaft	0	1	1	1	0	0	0	0	0	0	0	0	0	0	0	0	0	0	0	0	0	0	0	0	0	0	0	0	0	1	1
Lead screw	0	0	0	1	1	1	1	1	1	1	1	1	1	1	0	0	0	0	0	0	0	0	0	0	0	0	0	0	0	1	1
Cylinder end of the slider	0	0	0	0	1	1	1	1	1	1	1	1	0	0	0	0	0	0	0	0	0	0	0	0	0	0	0	0	0	1	1
Piston end of the slider	0	0	0	0	0	0	1	1	1	1	1	1	1	1	0	0	0	0	0	0	0	0	0	0	0	0	0	0	0	1	1
Link-1 (*d*_1_)	0	0	0	0	0	0	0	0	0	0	0	0	0	0	1	1	1	1	0	0	0	0	0	0	1	1	0	0	0	1	1
Link-2 (*d*_2_)	0	0	0	0	0	0	0	0	0	0	0	0	0	0	0	0	1	1	1	1	1	1	0	0	1	1	0	0	0	1	1
Link-3 (*d*_3_)	0	0	0	0	0	0	0	0	0	0	0	0	0	0	0	0	0	0	0	1	1	1	1	0	1	1	0	0	0	1	1
Link-2 (*d*_4_)	0	0	0	0	0	0	0	0	0	0	0	0	0	0	1	1	1	1	1	1	1	1	1	1	1	1	1	0	0	1	1
Rotary actuator- 1 (RA-1)	0	0	0	0	0	0	0	0	0	0	0	0	1	1	1	1	0	0	0	0	0	0	0	0	1	0	0	1	1	1	1
Rotary actuator- 2 (RA-2)	0	0	0	0	0	0	0	0	0	0	0	0	0	0	0	0	0	0	0	0	0	0	0	0	0	0	0	1	1	1	1

## Data Availability

Data are contained within the article.
